# Three Voices of the Atrium: A Unique Case Finding on a Neonate ECG

**DOI:** 10.1016/j.jaccas.2026.109437

**Published:** 2026-07-29

**Authors:** Qian Zhang, Hongguang Wu, Ying Huang, Haiying Li

**Affiliations:** Cardiovascular Medicine Center, Arrhythmia Department, The University of Hong Kong Shenzhen Hospital, Shenzhen, Guangdong Province, China

**Keywords:** acid-base imbalance, electrocardiogram, supraventricular arrhythmias

## Abstract

**Case Summary:**

We report a rare case of transient triple atrial beats observed in a full-term neonate presenting with severe metabolic acidosis (pH 7.097). The electrocardiographic findings revealed 3 distinct and electrically isolated atrial rhythms (P′, P″, and sinus P waves), which generated atrial fusion waves upon coincidental temporal overlap.

**Take-Home Message:**

Transient triple dissociated atrial rhythms are induced by severe metabolic acidosis and carry a favorable prognosis.

## Graphical Abstract

**Figure:**
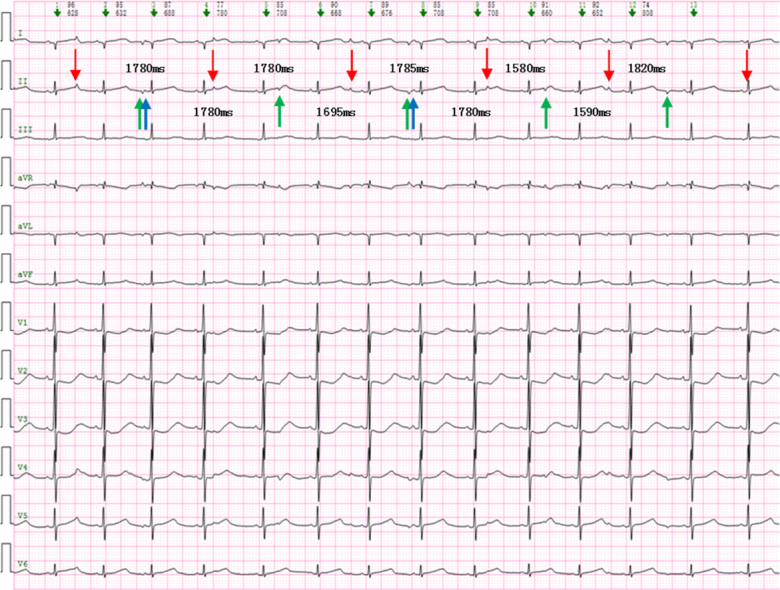

A full-term male neonate was born via spontaneous vaginal delivery, with multiple high-risk perinatal exposures including a short umbilical cord, grade III meconium-stained amniotic fluid, prolonged premature rupture of membranes, intrapartum late decelerations, and severe fetal bradycardia. He maintained excellent clinical transition with Apgar scores of 9, 10, and 10 at 1, 5, and 10 minutes. He was admitted to the neonatal intensive care unit because of metabolic acidosis. Umbilical artery blood gas analysis revealed a pH of 7.097. Bedside echocardiography revealed normal cardiac structure, preserved left ventricular systolic function (ejection fraction: 78%), and a small 1.9-mm patent foramen ovale with left-to-right shunt and trivial tricuspid regurgitation, without major congenital cardiac defects. Shortly after birth, bradycardia was observed, prompting a 12-lead electrocardiogram (ECG) (Figure 1).

**Take-Home Message**

• Transient triple dissociated atrial rhythms are induced by severe metabolic acidosis and carry a favorable prognosis.

**Figure 1:**
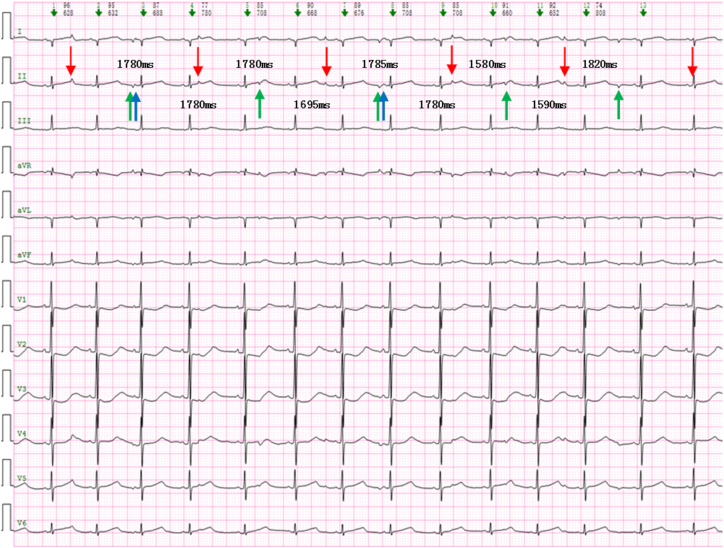
ECG Demonstrating a Ventricular Rate of Approximately 74 to 95 beats/min With Each QRS Complex Preceded by a P-Wave That Is Inverted in Lead aVR and Upright in Leads I, II, III, aVL, aVF, and V_2_-V_6_ The PR interval is constant, and the QRS complex exhibits supraventricular morphology with mild right-axis deviation and counterclockwise rotation. ST-segments in leads V_1_-V_3_ show a 0.10 mV downsloping depression, while T waves in all precordial leads (V_1_-V_6_) are upright. P′ waves (red arrow, cycle length 1,580-1,820 ms) and P″ waves (green arrow, cycle length 1,590-1,780 ms) demonstrate no fixed relationship with QRS complexes, each generating electrical impulses independently at stable cycle lengths unaffected by the atrial refractory period. Atrial fusion waves are also observed (blue arrows). ECG = electrocardiogram.

What diagnosis could explain this patient's polymorphic P-wave morphologies?

A. Nonconducted atrial premature contractions
B. Atrial dissociation
C. Atrial parasystole
D. Recording artifacts

The correct answer is B.

## Discussion

**Figure 2:**
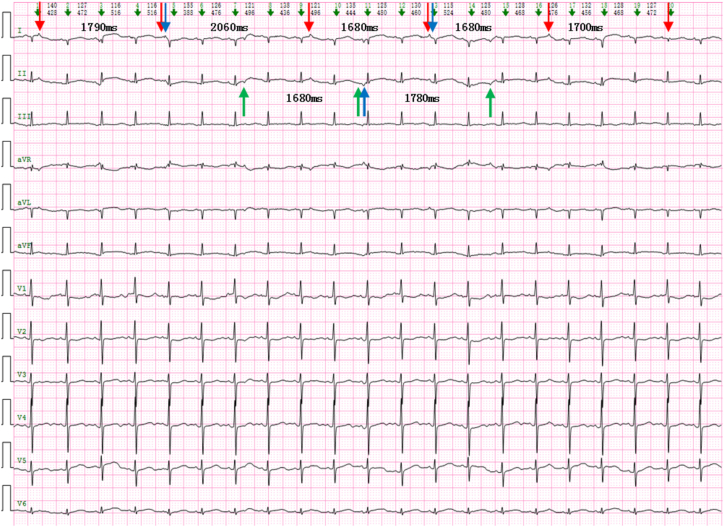
12-Lead ECG Recorded on the Second Day Postnatally Demonstrates a Dominant Sinus Rhythm With Regular RR Intervals and a Heart Rate of Approximately 125 beats/min The QRS complexes persistently exhibit right-axis deviation combined with counterclockwise rotation. Both P′ waves (red arrows, cycle length 1,680-2,060 ms) and P″ waves (green arrows, cycle length 1,680-1,780 ms) demonstrate complete dissociation from ventricular activity, maintaining independent rhythms unaffected by atrial refractory periods. Atrial fusion waves are identified at locations marked by blue arrows. While P′ waves display a regular occurrence pattern, P″ waves exhibit notable irregularity and intermittent activation. ECG = electrocardiogram.

**Figure 3:**
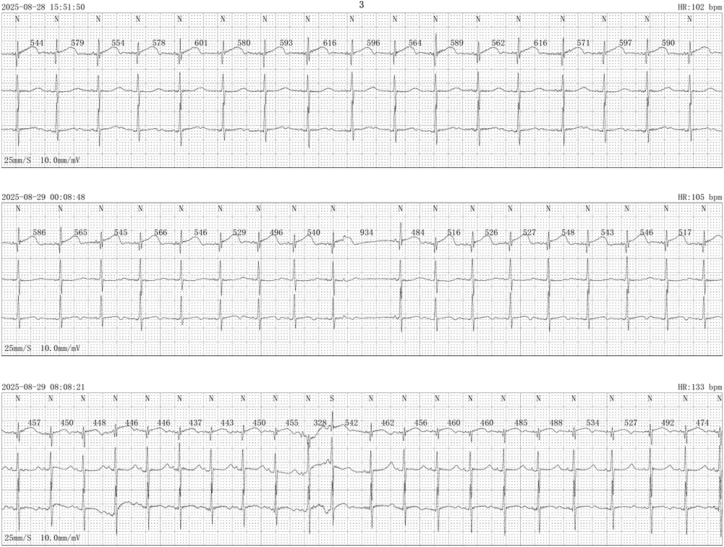
Three-Lead Synchronized Holter Monitor Recording Obtained on the Afternoon of the Second Postnatal Day Displays Leads MV5, MV1, and MaVF From Top to Bottom The 24-hour Holter recording documented 9 premature atrial contractions (PACs) in total. Representative segments from various monitoring periods consistently exhibited predominantly regular sinus rhythm, interspersed with sporadic isolated atrial premature beats. Notably, a portion of these ectopic beats manifested as nonconducted PACs followed by incomplete compensatory pauses.

Atrial dissociation provides the most plausible explanation for the observed ECG patterns. Two additional sets of dissociated atrial activity are observed: P′ waves (marked by red arrows in Figure 1) and P″ waves (green arrows) maintain complete electrical independence from both the sinus rhythm and each other. These ectopic atrial rhythms fire at their intrinsic rates without interference from atrial refractoriness. When P′ and P″ waves fortuitously coincided, they generated characteristic atrial fusion complexes (blue arrows), pathognomonic for atrial dissociation. A follow-up ECG performed on the second postnatal morning (Figure 2) revealed persistent atrial dissociation, evidenced by dual P′ (red arrows) and P″ (green arrows) wave activities. The P′ waves exhibited regular periodicity, whereas the P″ waves demonstrated intermittent irregularity. Subsequent 3-lead Holter monitoring later that afternoon documented 9 premature atrial contractions (PACs), including several nonconducted PACs accompanied by incomplete compensatory pauses (Figure 3). Notably, neither P′ nor P″ wave activity was detectable during this Holter recording period.

Atrial dissociation on ECG is characterized by a unilateral ectopic atrial rhythm that is independent of the primary rhythm, which can be sinus or ectopic.^1^ This rare electrophysiological phenomenon predominantly occurs in heart transplantation recipients and post–cardiac surgery patients,^2^, ^3^, ^4^ with fewer cases reported in digitalis toxicity or congestive heart failure.^5^ In the past, it was thought to be a marker of critical illness and high risk of imminent death.^1^ Recent studies indicate it may also present asymptomatically.^6^^,^^7^

While the ectopic atrial rhythm is usually slow, it may occasionally present as tachycardia, flutter, or fibrillation, with none of its impulses conducting to the ventricles.^1^ Our case exhibited the classic bradycardic pattern. Although dual-rhythm atrial dissociation has been previously documented in literature, this represents, to our knowledge, the first reported instance of transient triple atrial dissociation in a neonatal patient.

The diagnosis of atrial dissociation required careful differentiation from nonconducted atrial premature contractions (APCs) and atrial parasystole. The observed P′ and P″ waves were distinguishable from typical APCs by the absence of sinus P-wave interference and lack of consistent coupling intervals. Atrial parasystole is defined by protective entrance block around the ectopic focus with intermittent exit conduction capable of capturing the atria and resetting sinus rhythm, while the ectopic atrial activity in atrial dissociation is encircled by complete bidirectional intra-atrial block, precluding any exit conduction to the remainder of the atria or ventricles.^1^ In our case, the ectopic P′ and P″ waves demonstrated no fixed coupling with sinus P waves, never conducted to the ventricles even when falling in the nonrefractory period, and showed no evidence of sinus rhythm interruption, all of which are inconsistent with the classic electrocardiographic criteria of atrial parasystole.

The precise etiology of atrial dissociation in this case remains undetermined, particularly regarding the pathophysiological mechanism of its spontaneous resolution. We suspect that atrial dissociation in this patient may be attributed to metabolic acidosis. Acidosis induces a pH-dependent reduction in Connexin 43 (Cx43) conductance, resulting in gap junction channel closure, slowed conduction velocity, altered cellular excitability and refractoriness, and progressive electrical uncoupling.^8^, ^9^, ^10^ The heterogeneous electrical propagation between cardiomyocytes can result in localized conduction blocks or delayed activation.

Serial ECG monitoring demonstrated dynamic evolution correlating with pH normalization. By the second postnatal morning, one ectopic rhythm stabilized, while the other showed suppressed automaticity. Subsequent Holter monitoring (second postnatal afternoon) confirmed complete resolution of both P′ and P″ waves. The patient met discharge criteria after 6 days, with favorable outcomes.

## Funding Support and Author Disclosures

The authors have reported that they have no relationships relevant to the contents of this paper to disclose.
